# Optimization and impact of an evidence-based pre-audit prescription decision system in primary healthcare settings

**DOI:** 10.3389/fphar.2025.1491810

**Published:** 2025-04-14

**Authors:** Xiao-Hui Yue, Lei Yang, Jing-Jing Zhong, Hong-Mei Liu, Dan Wang, Xue Tao, Gao-Feng Zheng

**Affiliations:** ^1^ Department of Pharmacy, The People’s Hospital of Jianyang City, Chengdu, China; ^2^ Department of Pharmacy, Sichuan Academy of Medical Sciences and Sichuan Provincial People’s Hospital, School of Medicine, University of Electronic Science and Technology of China, Chengdu, China; ^3^ Department of Infection, Ziyang Central Hospital, Ziyang, China

**Keywords:** medication errors, pre-prescription review, prescription pre-audit intelligent decision system, evidence-based practice, rational drug use

## Abstract

**Objective:**

Analyze the operation mode of the prescription pre-audit intelligent decision system in a county-level hospital, evaluate its intervention effects on outpatient and emergency operations, thus providing references for similar hospitals to carry out pre-audit intelligent decision system and promote rational drug use.

**Methods:**

Utilizing evidence-based approaches, system rule modifications have been refined and synergized with AI-driven decision-making analytics to examine the operational framework of pre-audit prescription decision system. Additionally, retrospectively analyze the types and levels of problems triggered by outpatient and emergency prescriptions from October 2022 to August 2023, as well as the rationality of prescriptions in the system.

**Results:**

According to the clinical operation of the hospital, problems triggered by unreasonable prescriptions have been finely classified into different levels according to the severity of prescription problems. From October 2022 to August 2023, the number of prescriptions triggering issues such as indications, dosage, special populations, compatibility, administration, and contraindications showed a decreasing trend compared with October 2022 before the intervention. For example, the number of prescriptions with unreasonable routes of administration decreased from 1,745 to 20, and the number of contraindicated prescriptions decreased from 1,399 to 16. The prescriptions triggering Level 5 alerts decreased from 5.609% to 1.793% and the prescription compliance rate increased from 92.20% to 95.98%.

**Conclusion:**

The prescription pre-audit intelligent decision system enhances patient safety and promotes rational drug use. However, the system requires fine-tuning and continuous improvement of the system rule library to effectively validate prescriptions and improve prescription accuracy. In the future, integrating big data, artificial intelligence and other technologies for secondary system development will be a model worthy of consideration. In addition, promoting this system to medical federation to establish a regional prescription review model will further promote the high-quality development of pharmaceutical services.

## 1 Introduction

Medication errors may occur at any stage of patient medication use, such as when a doctor prescribes medication, a pharmacist dispenses medication and other actions by hospital personnel (Pais et al., 2024; Bowdle et al., 2023; Subramanian et al., 2022). Globally, medication errors cause 5%–41.3% of all hospitalizations and 22% of readmissions after discharge (Tariq et al., 2024). The annual cost of treating patients affected by medication errors exceeds $40 billion (Wang et al., 2022). Additionally, a total of 27,460 cases of medication error were collected in the China National Monitoring Network for Clinical Safe Medication in 2023, of which the main people who triggered by physicians (19,655, 71.58%) and pharmacists (5,688, 20.71%) (Zhang et al, 2024a). This greatly impacts patients’ health and hospitals’ reputation.

In most grassroots hospitals in China, patients typically go to the pharmacy to collect medication after the doctor has issued a prescription, and then the pharmacist dispenses the medication after verifying the prescription information. Creating prescriptions is an early step in patients’ medication process. As prescription errors are common and preventable medication errors, pharmacists’ review of prescriptions is crucial for detecting errors and preventing patient adverse effects (Ben Natan et al., 2017; Lee et al., 2022; Bu et al., 2022). However, due to the large number of prescriptions and a shortage of pharmacists in Chinese hospitals, there may be omissions during pharmacist review of prescriptions (Zhao et al., 2021). Additionally, due to differences in the professional abilities of each pharmacist, there is a lack of consistency in prescription review. Currently, the informatization and intelligent construction of hospitals in China are being vigorously promoted (Wang et al., 2024). Against the backdrop of new policies, the development of patient-centered hospital pharmacy requires more refined informatization systems as support, while also placing higher demands on hospital pharmacy services (Young et al., 2023; Allen et al., 2024a). Therefore, embedding intelligent rational drug use software in hospital information systems to intelligently review prescriptions when doctors create prescriptions, which may effectively avoid prescription errors (Westbrook et al., 2022; Pereira et al., 2023).

In recent decades, the gradual advancement of Health Information Systems (HIS) has significantly enhanced patients’ medical experiences and the quality of care (Tamrat et al., 2022; Tummers et al., 2021; Seth et al., 2024). Electronic medical records, electronic prescribing, and artificial intelligence-based decision support systems are pivotal in refining HIS (Nimri et al., 2024). The Prescription Pre-Audit Intelligent Decision System (PPIDS) is a rational medication support tool integrated into our HIS (Wang et al., 2022; Kassem et al., 2024). It is designed to provide intelligent decision support to physicians during medication prescribing, aiming to effectively reduce prescription errors. This system is constructed and continuously updated based on current drug instructions, guidelines, and peer-reviewed research (Wang et al., 2022; Li et al., 2022). Consequently, it assesses drug usage information when physicians issue prescriptions, intervening in inappropriate prescriptions according to varying safety levels (Westbrook et al., 2022). This process helps identify prescription errors early, enhances the consistency of medical practices, and promotes medication safety. Despite the integration of intelligent decision support in hospital systems, there is limited research evaluating its real-world effectiveness in reducing prescription errors at county-level hospitals. This study aims to bridge that gap by assessing the impact of PPIDS in clinical practice.

## 2 Methodology

### 2.1 The prescription pre-audit intelligent decision system

**FIGURE 1:**
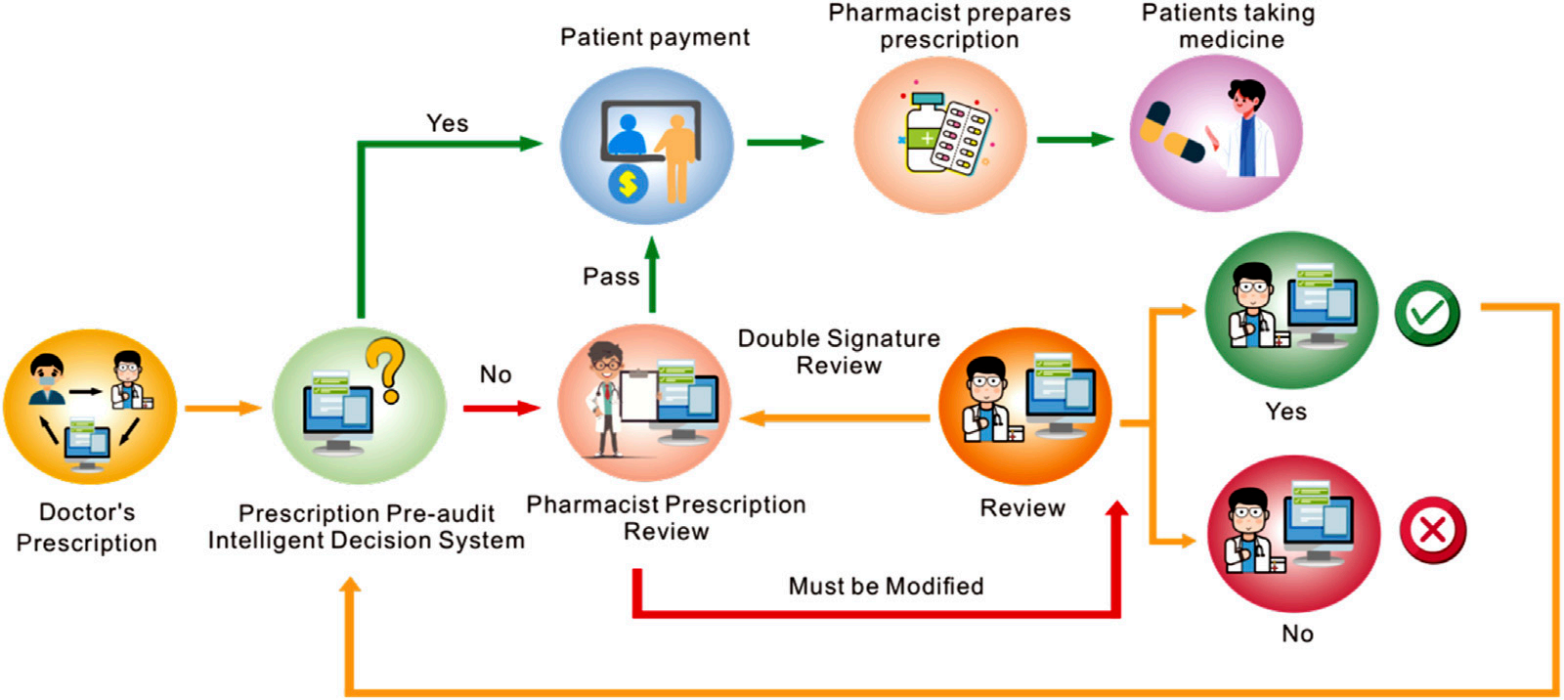
Operation of the Pre-audit Intelligent Decision System. In essence, all prescriptions issued to patients following a medical consultation must be reviewed by the Pre-audit Intelligent Decision System. If a prescription is approved, the patient can proceed with payment and receive the medication. If a prescription is not approved, it must be either double-signed or modified; otherwise, it cannot be processed.

This study was conducted at the People’s Hospital of Jianyang City, which is a comprehensive tertiary grassroot hospital in Sichuan, China (Figure 1). The PPIDS was designed by Hangzhou Yiyao Co., Ltd. The system was embedded into the HIS, followed by system debugging and integration. Subsequently, the system underwent silent operation without prompts or interventions to collect debugging data. The collected trigger rule information was organized, and pharmacists retrieved relevant drug instructions, guidelines, and online databases (such as Micromedex, Uptodate, Drugs) based on the triggered drug rule information. An intelligent prescription knowledge database was constructed and embedded into the PPIDS to provide drug rule suggestions (i.e., different levels). Finally, after communication and discussion with doctors, the system began to operate (Supplementary Figures 1, 2).

### 2.2 Warning levels of system

**FIGURE 2:**
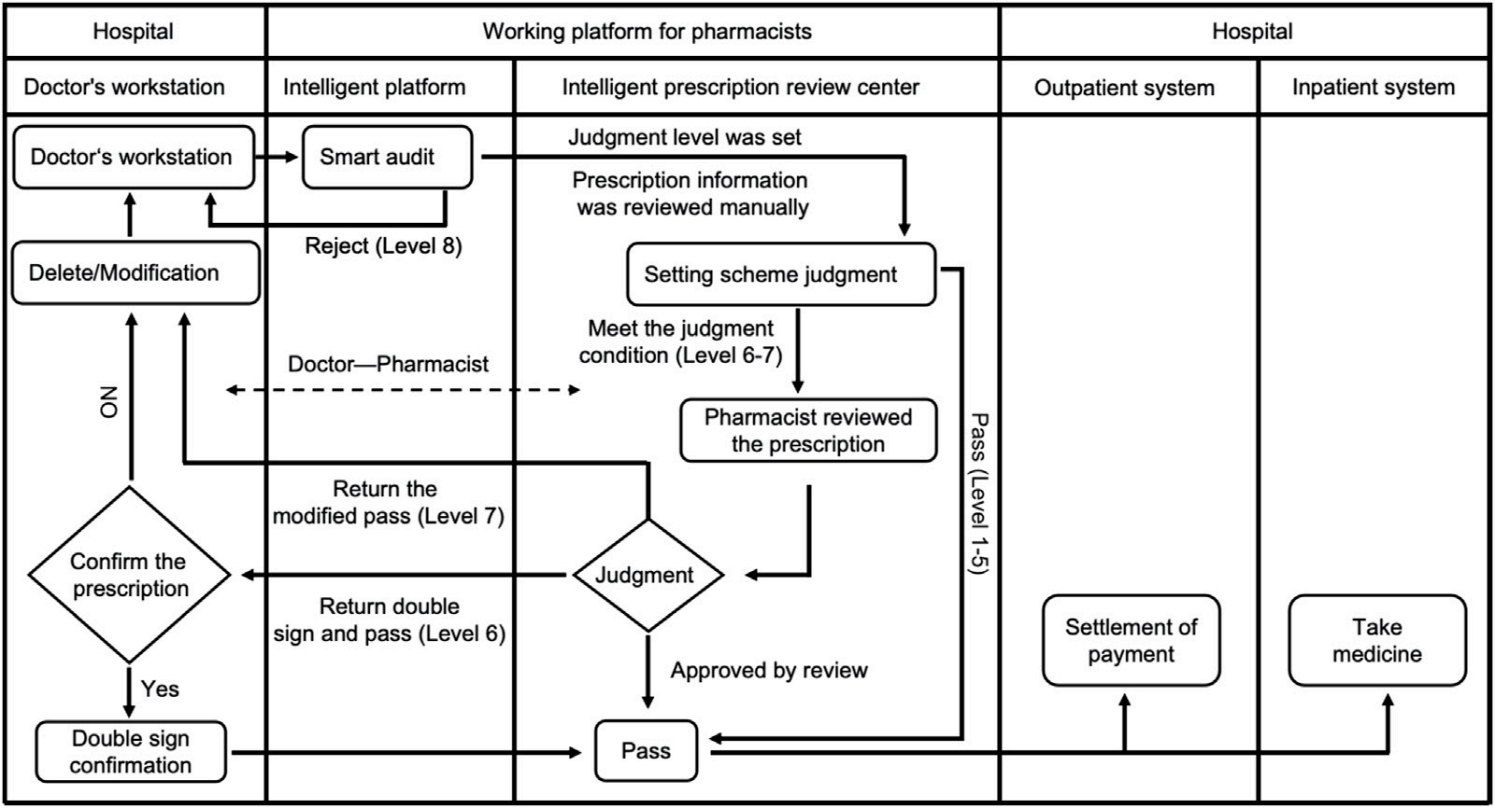
Flow chart for prescription review. During the prescription issuing process, the Prescription Pre-audit Intelligent Decision System automatically identifies the medication information prescribed by the doctor, evaluates its appropriateness, and conducts a review. Pre-audit of medical orders mainly includes steps: processing prescriptions based on different warning levels; when Levels 6 or 7 warnings appear, pharmacists conduct manual reviews of prescriptions, while prescriptions with Level 8 warnings cannot be issued. In summary, if there are no errors in the medical orders or prescriptions issued by the doctor, the medication will be successfully packaged and dispensed. Otherwise, it will not pass.

All prescriptions are issued by physicians and stored in the electronic medical record system. Prescription orders are automatically sent to the prescription PPIDS for review. Drug alert messages are categorized into levels 1–8 based on literature review, consensus guidelines, and clinical discussions. Briefly, levels 1–4 are advisory messages (not shown to physicians, e.g., downgraded from higher alert levels after clinical discussion and evidence collection). Level 5 warnings are advisory messages shown to physicians (indicating potential medication error risks, e.g., “unclear,” “use with caution,” “weigh risks and benefits”). Level 6 warnings require dual signing by physicians (physicians must confirm before saving, e.g., severe hepatic or renal impairment requiring clinical assessment before use). Level 7 warnings require modification by physicians (system identifies prescription errors that need correction before saving, e.g., inappropriate dosage for traditional Chinese medicine, inappropriate administration frequency). Level 8 warnings prevent physicians from submitting prescription information (clear errors preventing prescription issuance, e.g., exceeding maximum dosage, obvious route of administration errors) (Figure 2). It is of significance to note that the false positive alerts caused by problematic rules must be treated with prudence. After obtaining rigorous evidence-based proof, it is essential to modify the rules to ensure their authority and effectiveness (Supplementary Figure 2). On the premise of guaranteeing the rational use of medications by patients, this also serves to prevent the addition of unnecessary prompts in the clinical setting.

### 2.3 Study design and data collection

This study is a retrospective study that collected outpatient and emergency prescriptions at levels 5–8 were collected from Jianyang People’s Hospital from October 2022 to August 2023. Data were collected and exported using the Hangzhou Yiyao Rational Drug Use Management System (Hangzhou Yiyao Co., Ltd.) for analysis and processing. Pharmacists conducted to double check on the safety of medications, and then responded according to different warning levels (Wang et al., 2022). This study is mainly a retrospective descriptive analysis, focusing on orders that evoke alerts, with detailed information that was collected and entered into Microsoft Excel 2024 and GraphPad 9.0 software for further analyses. The trend of data changes was compared with the initial observation data (October 2022), and the pre-intervention data (October 2022 to March 2023) and post-intervention data (April 2023 to August 2023) were statistically analyzed before and after. The unpaired sample t-test was used to compare the means of the two groups, and the Wilcoxon test of paired samples was used for non-normally distributed data. A two-sided P < 0.05 was considered statistically significant.

### 2.4 Ethics

This study protocol was approved by the Ethics Committee of Jianyang People’s Hospital (ethical approval number: JYL2024004Z). As it is a retrospective study, written form informed consent was not required.

## 3 Results

### 3.1 Analysis of prescription problems

**FIGURE 3:**
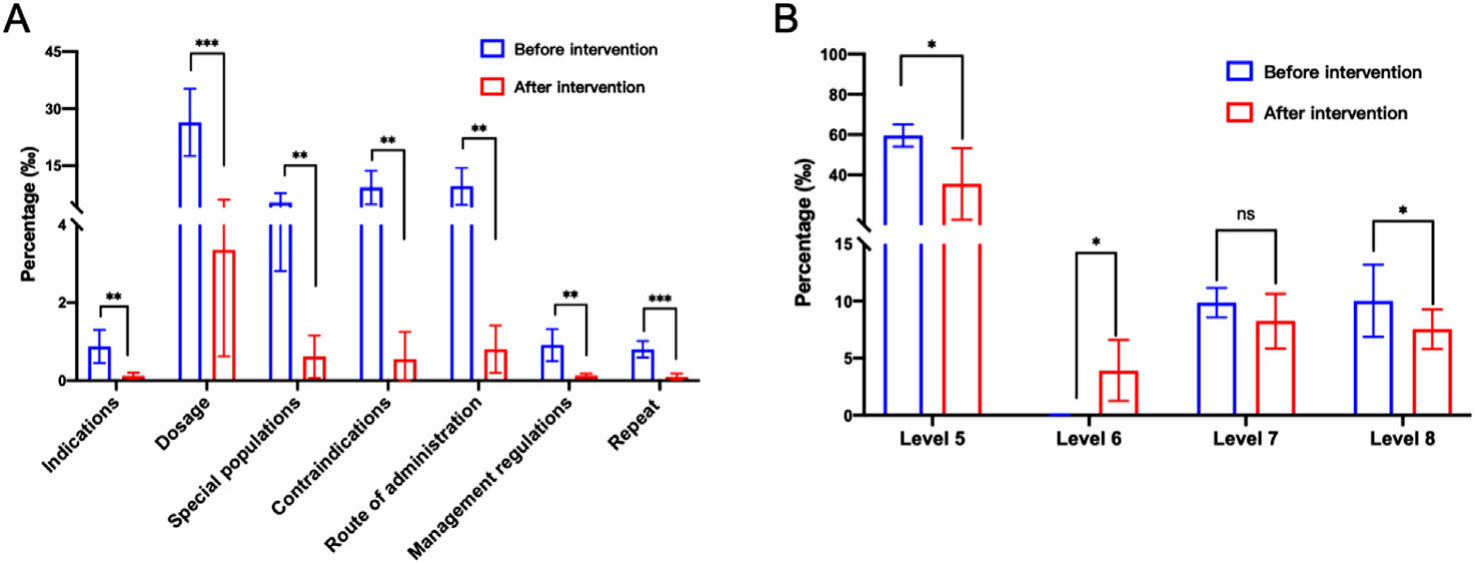
Pre-audit Intelligent Decision System intervention alert types and levels. **(A)** Pre-audit Intelligent Decision System intervention alert types. **(B)** Pre audit Intelligent Decision System intervention alert level 5 -8. Statistical significance was calculated via t-test, *p < 0.05; **p < 0.01; ***p < 0.001.

Collect data of rule-triggered type after the system goes live for analysis (Supplementary Table 1). From October 2022 to August 2023, there has been a general decrease in problematic prescriptions in terms of indications, dosage, special populations in outpatient and emergency prescriptions (Supplementary Figures 3A, B). For example, in October 2022, the number of prescriptions with dosage issues was 3,588, accounting for 2.912% of that month. By August 2023, the number decreased to 83, representing 0.071% of that month, a decrease of 2.841%. It is worth noting that there have been significant changes in the administration route and contraindications during this period (Figure 3A), decreasing from 1,745 to 20, and from 1,399 to 16, respectively. This change may be attributed to the initial in the system rules, which have been subsequently improved and modified in the rule library. Moreover, the frequency of alerts for most drugs exhibited a decline from March to August 2024. This phenomenon may be attributable to the refinement of our algorithm system, which consistently prompted physicians to improved prescribing behavior (Supplementary Table 2). Therefore, grounded in evidence-based medicine, the continuous enhancement of the system rule library in integration with clinical practice assumes a pivotal role in guaranteeing the rational utilization of medications and the seamless operation of the system (Ibáñez-Garcia et al., 2019; Tantray et al., 2024).

### 3.2 Level analysis of triggering questions

By collecting data through the system, different alert levels are classified (Supplementary Table 3). In October 2022, the trigger rate of level 5 warning in the system rule base is 5.609%. As the system rule library continues to be improved, the rules are gradually classified into different levels to achieve different warning effects. As of August 2023, the triggering rate of level 5 alerts has decreased to 1.793%, while level 6 alerts have significantly increased (Figure 3B; Supplementary Figure 3C). This suggests that through continuous improvement of the system and fine-grained classification of levels, using different alert levels not only increases doctors’ attention to prescription standards but also avoids ineffective pop-up alerts, thereby facilitating rational medication use for patients.

### 3.3 Analysis of prescription rationality

**FIGURE 4:**
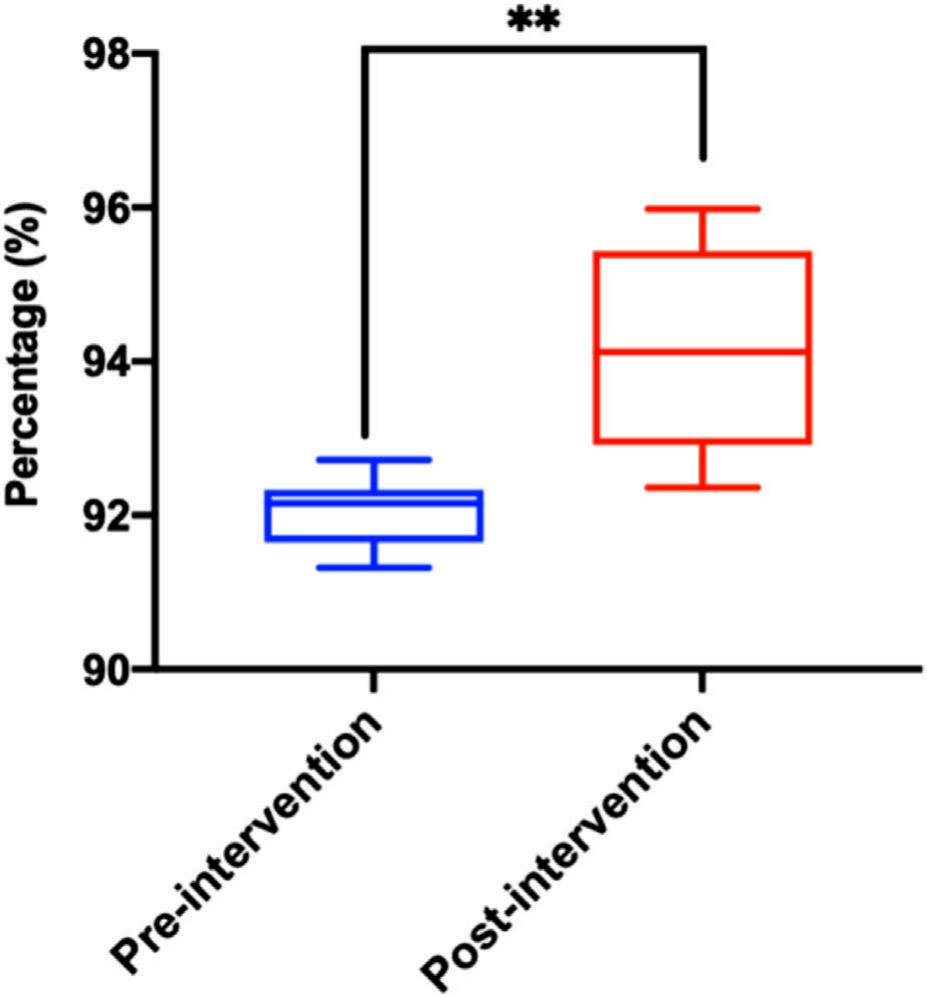
Distribution of reasonable prescription rate after Pre-audit Intelligent Decision System intervention. Statistical significance was calculated via t-test, *p < 0.05; **p < 0.01; ***p < 0.001.

The rationality of prescriptions is significant importance in safeguarding patient safety, enhancing treatment efficacy and preventing drug abuse. The warning levels in this study are classified into levels 1–8. Levels 1–4 do not indicate any warning, and levels 5 and above may involve potential medication errors or even worse. Therefore, we define levels 5–8 as inappropriate medication prescriptions and further infer rational medication prescriptions. By analyzing the system data exported from PPIDS from October 2022 to August 2023, the results show a gradual increase in the prescription rationality rate (Table 1; Figure 4; Supplementary Figure 3D). Noteworthy, the system determined the prescription rationality rate to be 91.32% in January 2023. This is due to the tight supply of drugs and the severe epidemic situation of COVID-19 in China in early 2023, which led to the restriction of related drugs in short supply, resulting in an increase in alerts triggered by doctors and a significant decline in the rationality of prescriptions. Subsequently, with rapid adjustments and the introduction of relevant management policies, the prescription rationality rate gradually improved. Additionally, the system’s rationality rate may be subject to false negatives and false positives. Therefore, further analysis of the rationality of prescriptions prompted by the system needs to be conducted in conjunction with clinical practical issues and cannot be generalized.

**TABLE 1:** Summary of rational rate of outpatient and emergency prescriptions in hospital.

	Time	Total number of prescriptions	Unreasonable number of prescriptions	Reasonable number of prescriptions	Reasonable rate (%)
Pre-intervention	2022.10	123,231	9,609	113,622	92.20%
	2022.11	121,274	8,832	112,442	92.72%
	2022.12	165,880	13,011	152,869	92.16%
	2023.01	99,534	8,635	90,899	91.32%
	2023.02	102,769	8,072	94,697	92.15%
	2023.03	149,107	12,287	136,820	91.76%
Post-intervention	2023.04	127,239	9,720	117,519	92.36%
	2023.05	137,301	8,960	128,341	93.47%
	2023.06	130,296	7,656	122,640	94.12%
	2023.07	121,105	6,190	114,915	94.89%
	2023.08	117,271	4,710	112,561	95.98%

## 4 Discussion

Our study indicates that PIDDS can providing real-time prescription feedback to physicians through intelligent review can reduce medication errors. This integration offers substantial benefits for enhancing diagnostic and treatment plans. For instance, the system can mitigate medication errors by alerting physicians with warning information about drugs (Supplementary Table 2). Among these alerts, dosage-related warnings are a frequent cause of medication errors, consistent with findings from a study conducted in Denmark (Rishoej et al., 2017). Furthermore, configuring abnormal dosage alerts within the system is crucial for preventing medication errors due to physician oversight (Wu et al., 2020).

It is noteworthy that incorrect routes of administration were the second type of error in this study, accounting for 1.416% of all prescription that month. Variations in drug preparation processes can lead to different routes of administration for the same medication (Liu et al., 2023). Errors in the route of administration can have serious adverse effects on patient safety (Gualano et al., 2021), such as prescribing “oral” medications as “nebulized” or “topical” medications as “oral,” among other issues. In this study, 1,745 alerts for “route of administration” errors were triggered in October 2022; however, this number decreased to 20 by August 2023 (Supplementary Table 1). This reduction may be attributed to initial discrepancies between the HIS’s “routes of administration” and at the time of system installation. Additionally, as medications are continually updated, ongoing system optimization is necessary.

In this study, medication errors are more prevalent in the hospital’s convenience outpatient clinics. In August 2023, the highest number of rule-triggered alerts of severity level 7 or above were recorded, accounting for approximately 4.82% of the total alerts (Supplementary Table 4). This trend may be attributed to grassroot hospitals offering services in non-specialized departments to accommodate patients’ needs for convenient access to care. Consequently, prescribing physicians, who may lack familiarity with specialized medications, are more likely to trigger medication warnings. Nevertheless, this observation underscores the critical role of the warning system in reducing the likelihood of medication errors. Notably, in cases involving critical care patients with clinical pharmacist involvement, only 6 alerts of level 7 or above were triggered, representing approximately 0.13% of the total (Supplementary Table 4). This contrasts with findings from Alghamdi et al. (2019). And underscores the significant role of clinical pharmacists in clinical practice and their contribution to assisting physicians in managing patient care.

The “Management Regulations” governing the review system are commendable, which are not only derived from hospital-specific policies but also from government guidelines. By embedding PPIDS in a structured manner, they play an important role in hospital drug and prescription management. They standardize physician prescribing practices and enhance the rationality of prescriptions. Furthermore, these regulations facilitate the dissemination of pertinent policies and guidelines to frontline clinicians, thereby aiding the execution of related tasks. Additionally, the “Management Regulations” provisions ensure drug supply under exceptional circumstances. For instance, during the COVID-19 pandemic, when drug production was insufficient to meet demand, regulations such as restricting acetaminophen prescriptions to 10 tablets per person per prescription were implemented to better ensure patient access to essential medications.

In recent decades, remarkable technological progressions have opened novel avenues for safeguarding patient safety (Li et al., 2024; Kurniawan et al., 2024). The integration of technology to digitize healthcare processes the potential to enhance the standardization and efficiency of clinical work processes, which mitigate medication errors across all medical institutions. Specifically, the shift from paper-based prescriptions to commercial computerized provider order entry (CPOE) systems has been demonstrated to reduce MEs and hospital mortality rates (Holmgren et al., 2023). The utilization of effective CPOE and clinical decision support system (CDSS) can avert or decrease medication errors (Ciapponi et al., 2021). One study showed that well-implemented CPOE systems led to a significant reduction in both serious and common prescription-related and procedural errors (Sheikh et al., 2022). However, systems with suboptimal designs are ill-suited to existing workflows, which may engender user dissatisfaction and heighten the likelihood of errors (Bocknek et al., 2022). Consequently, it is imperative to continuously optimize the rules to align with the existing workflows.

With the emergence of artificial intelligence systems, integrating PPIDS with intelligent platforms plays an important role in facilitating prescription review in clinical practice (Toffaha et al., 2023; Zhang et al, 2024b; Mohsen et al., 2023). Currently, the updating of the review system’s rule database often requires extensive evidence-based collection and processing (Grammatikopoulou et al., 2024a), followed by discussions before further modifications can be made (Lester et al., 2021). This not only increases the workload of pharmacists and physicians, but also makes problem resolution more cumbersome (Grammatikopoulou et al., 2024b). Therefore, the application of machine learning and big data analysis processing systems becomes particularly important. Such as building interconnected data processing centers across multiple databases, machines automatically upgrade the system based on new instructions, guidelines, and other databases to achieve precise prompts. Additionally, the establishment of intelligent review centers is crucial for prescription review in grassroots hospitals and ensuring medication safety (Rojas Garcia and Antonanzas Villar et al., 2021).

The effective operation of the review system relies heavily on the refinement and optimization of the core rule database (Hegde et al., 2022). As AI-based systems continue to evolve, special medications require specific management settings to enhance patient medication safety (Allen et al., 2024b). Furthermore, management settings for off-label drug use, prescription rights, and emergency drug supply management are beneficial for fine-tuning clinical decision-making. Strengthening communication with clinical staff, providing continuous personnel training, and building an evidence-based database can promote to constructing a refined drug rule database.

Notably, the PPIDS not only efficiently enhances the efficiency of pharmacists’ prescription review via prior intervention, but also furnishes a basis for judgment in clinical drug management decision-making. Nevertheless, it still has deficiencies in data updating, refinement, and coverage. False alerts may engender inconvenience and necessitate continuous attention. Furthermore, the system regulates pharmacists’ prescription behavior through prompt alerts. The incessant pop-up box alerts may require optimization in the future, as they might trigger pharmacists’ resistance to the system. The principal limitation of this study was its single-center nature. This descriptive retrospective study was carried out in only one setting and may not be generalizable to large-scale healthcare settings in China. Another limitation is the dearth of in-depth analysis of the causes of these MEs and the absence of rigorous statistical analysis.

## 5 Conclusion

In summary, under the supervision of trained pharmacists, PPIDS effectively verifies prescriptions, thereby improving prescription accuracy and medication safety. The application of the PPIDS enables hospital pharmacists to conduct prescription reviews more efficiently and accurately. This is of substantial significance for the future development and construction of intelligent hospitals. Moreover, in the future, greater emphasis should be placed on the AI integration. The utilization of big-data models to conduct in-depth research on hospital prescription information will encounter both opportunities and challenges in the process of providing more intelligent and individualized solutions.

## Data Availability

The original contributions presented in the study are included in the article/Supplementary Material, further inquiries can be directed to the corresponding author.
