# Supplementary material for: Optimization and impact of an evidence-based pre-audit prescription decision system in primary healthcare settings
Source: Front Pharmacol. 2025 Apr 14;16:1491810. doi: 10.3389/fphar.2025.1491810 (PMC12034548; doi:10.3389/fphar.2025.1491810)
Supplement: Supplementary file 1 [file DataSheet1.pdf]

Optimization and Implementation of Evidence-based Prescription Pre-audit Intelligent Decision System in grassroot Hospitals

Figure. S1.

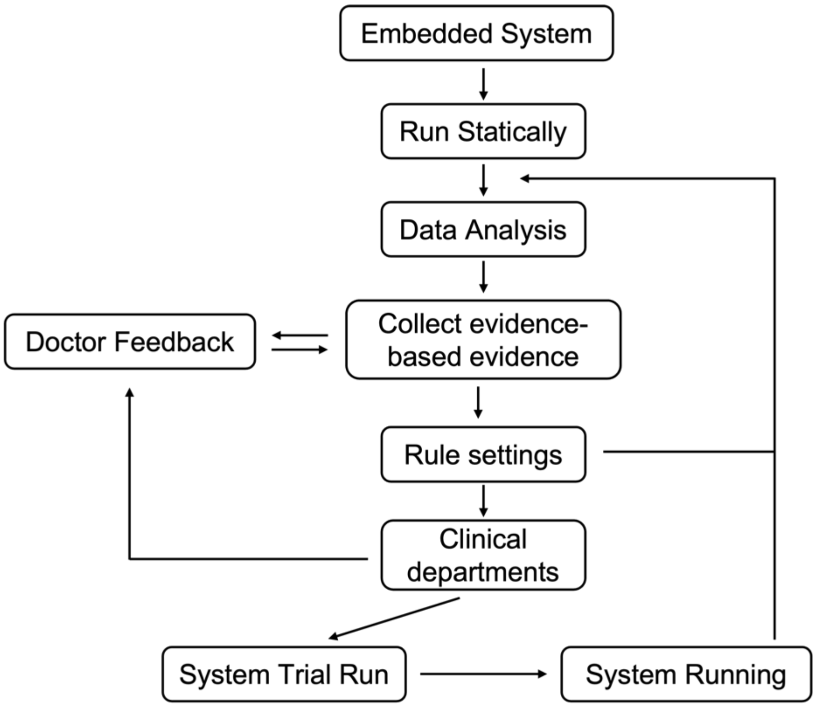

Supplementary Fig. 1 Pre-audit Intelligent Decision System embedded in the hospital management system process and related rules

Figure. S2.

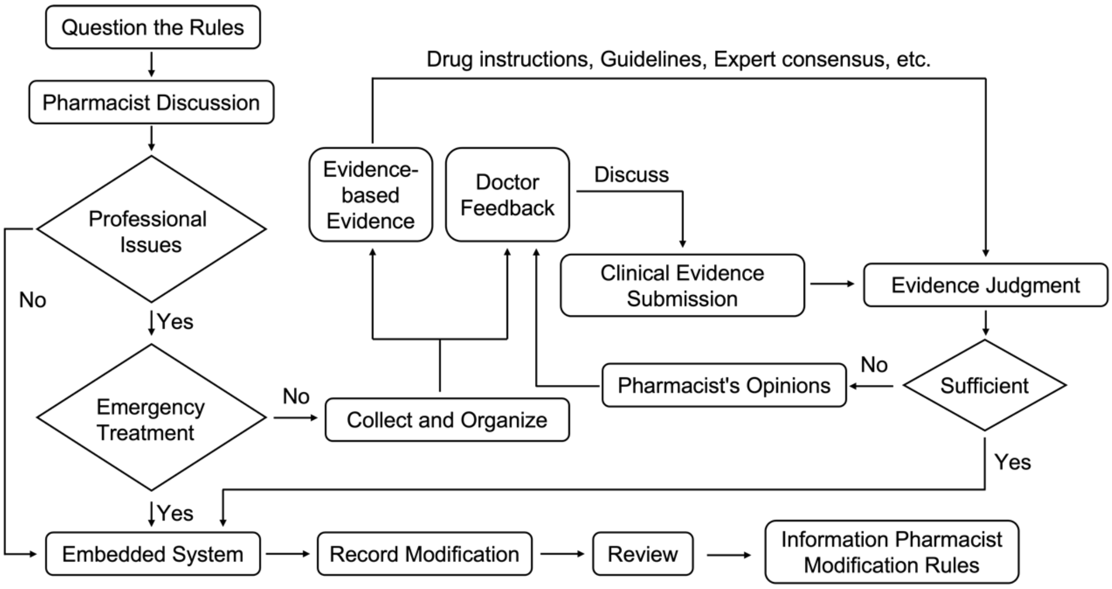

Supplementary Fig. 2 Rule modification of Pre-audit Intelligent Decision System

Figure. S3.

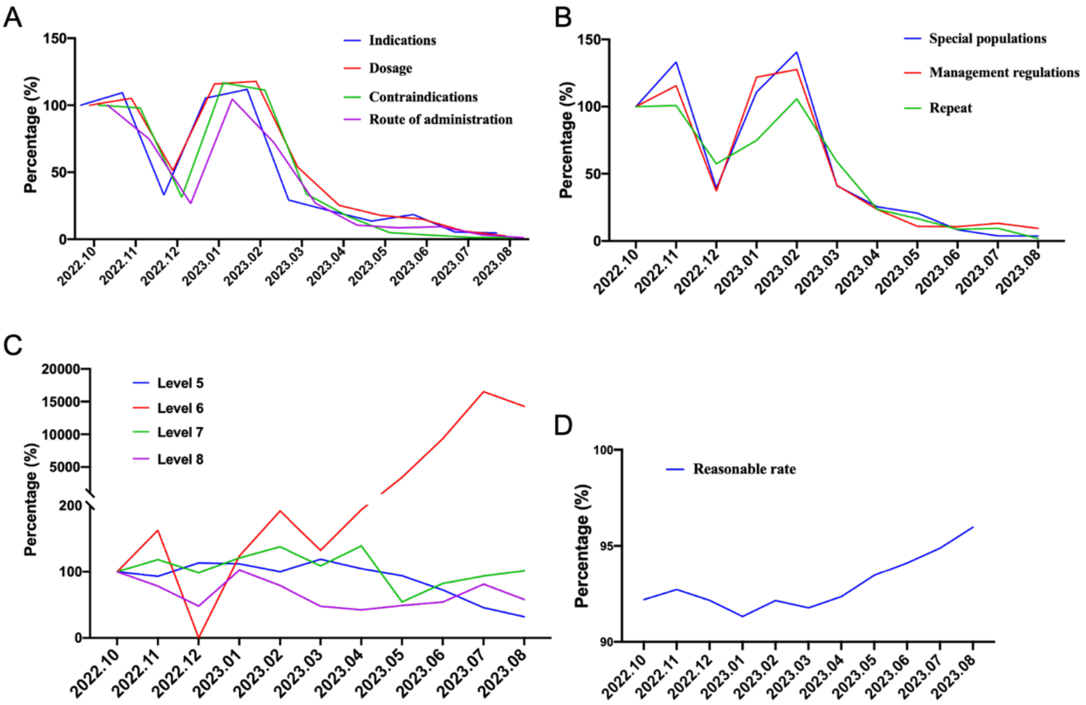

Supplementary Fig. 3 Prescription problems in outpatient and emergency departments data change trend chart. (A-B) Trend chart of changes in various prescription problems. (C) Trend chart of different levels of warnings triggered by the system. (D) Trend chart of prescription rationality rate.

Table. S1.

Supplementary Table 1. Summary of various types of prescription problems in outpatient and emergency departments of hospital

	Time	Total number of prescriptions	Indications		Dosage		Special populations		Contraindications		Route of administration		Management regulations		Repeat	
Pre-intervention	2022.10	123231	133	0.108%	3588	2.912%	694	0.563%	1399	1.135%	1745	1.416%	124	0.101%	119	0.097%
	2022.11	121274	143	0.118%	3712	3.061%	910	0.750%	1349	1.112%	1285	1.060%	141	0.116%	118	0.097%
	2022.12	165880	59	0.036%	2473	1.491%	366	0.221%	590	0.356%	625	0.377%	62	0.037%	92	0.055%
	2023.01	99534	113	0.114%	3356	3.372%	621	0.624%	1318	1.324%	1474	1.481%	122	0.123%	72	0.072%
	2023.02	102769	124	0.121%	3524	3.429%	814	0.792%	1298	1.263%	1050	1.022%	132	0.128%	105	0.102%
	2023.03	149107	47	0.032%	2338	1.568%	345	0.231%	570	0.382%	569	0.382%	62	0.042%	85	0.057%
Post-intervention	2023.04	127239	29	0.023%	936	0.736%	182	0.143%	247	0.194%	190	0.149%	30	0.024%	29	0.023%
	2023.05	137301	20	0.015%	711	0.518%	160	0.117%	78	0.057%	166	0.121%	15	0.011%	22	0.016%
	2023.06	130296	26	0.020%	567	0.435%	61	0.047%	45	0.035%	174	0.134%	14	0.011%	11	0.008%
	2023.07	121105	7	0.006%	214	0.177%	26	0.021%	19	0.016%	56	0.046%	16	0.013%	11	0.009%
	2023.08	117271	6	0.005%	83	0.071%	24	0.020%	16	0.014%	20	0.017%	11	0.009%	2	0.002%

Table. S2.

Supplementary Table 2. Based on the statistics for the number of times alert occurrences for drugs of Level 7 and Level 8 the top 20 on March 2024

**Drugs**	**Number of alert occurrences**					
	March 2024	April 2024	May 2024	June 2024	July 2024	August 2024
Roxithromycin Sustained Release Capsules	50	25	10	2	0	2
Oseltamivir Phosphate Granules	45	11	0	0	0	0
Insulin Aspart 30 Injection	33	31	0	0	0	0
30/70 MixtureRecombinant Human Insulin Injection	23	12	0	0	0	0
Estazolam Tablets	22	77	0	5	4	2
Hydrotalcite Chewable Tablets	22	12	10	7	4	4
Clonazepam Tablets	22	12	7	4	0	0
Insulin Degludec and Insulin Aspart Injection	20	31	0	0	0	0
Live Combined Bifidobacterium and Lactobacillus Tablets	19	0	0	0	0	0
Sulpiride Tablets	14	1	7	1	3	1
Compound Gargle Solution Chlorhexidine Giuconatie	7	0	0	0	0	0
Compound Glycyrrhiza Oral Solution	6	5	2	0	4	0
Huoxiang Zhengqi Koufuye	6	5	1	3	2	2
Promethazine Hydrochloride Tablets	6	5	5	0	1	0
Ganciclovir Ophthalmic Gel	5	0	0	0	0	0
Montelukast Sodium Chewable Tablets	5	4	1	0	0	0
Lacidophilin Tablets	5	1	7	17	1	2

Ketoprofen Gel	5	1	1	1	1	0
IbuprofenSuspension Drops	4	1	0	0	1	0
Insulin Glargine Injection	4	2	0	0	0	0

Table. S3.

Supplementary Table 3. Summary table of level 5-8 of alert occurrences in outpatient and emergency department of hospital

	Time	Total number of prescriptions	Level 5		Level 6		Level 7		Level 8	
Pre-intervention	2022.10	123231	6912	5.609%	5	0.004%	1066	0.865%	1626	1.319%
	2022.11	121274	6329	5.219%	8	0.007%	1241	1.023%	1254	1.034%
	2022.12	165880	10539	6.353%	8	0.005%	1413	0.852%	1051	0.634%
	2023.01	99534	6243	6.272%	5	0.005%	1040	1.045%	1347	1.353%
	2023.02	102769	5766	5.611%	8	0.008%	1224	1.191%	1074	1.045%
	2023.03	149107	9932	6.661%	10	0.007%	1402	0.940%	943	0.632%
Post-intervention	2023.04	127239	7467	5.868%	10	0.008%	1530	1.202%	713	0.560%
	2023.05	137301	7235	5.269%	191	0.139%	646	0.470%	888	0.647%
	2023.06	130296	5301	4.068%	494	0.379%	928	0.712%	933	0.716%
	2023.07	121105	3099	2.559%	812	0.670%	981	0.810%	1298	1.072%
	2023.08	117271	2103	1.793%	679	0.579%	1029	0.877%	899	0.767%

Table. S4.

Supplementary Table 4 Statistics of Level 7 and Level 8 alerts triggered by hospital departments in August 2024

Clinical Departments	Number of Level 7 and Level 8	Proportion
Convenient Clinic	227	4.82%
Gynecology	107	2.27%
Gastroenterology	93	1.97%
Cardiology I	87	1.85%
General Pediatrics	86	1.83%
Orthopedics	82	1.74%
Neurology	77	1.63%
Anesthesiology	70	1.49%
Oncology	69	1.46%
obstetrics	66	1.40%
Respiratory Medicine	60	1.27%
Traditional Chinese Medicine Orthopedics	60	1.27%
Nephrology	58	1.23%
Otolaryngology	56	1.19%
dermatology	55	1.17%
General Medicine	55	1.17%
Emergency Department	53	1.13%
Endocrinology	49	1.04%
Cardiology II	43	0.91%
Rheumatology	42	0.89%

Hematology	40	0.85%
General Surgery II	31	0.66%
General Surgery III	29	0.62%
Rehabilitation Department	25	0.53%
General Surgery I	24	0.51%
Dentistry	23	0.49%
Class A general ward	21	0.45%
Urology	21	0.45%
Psychosomatic	21	0.45%
Burns and Plastic Surgery	19	0.40%
Thoracic Surgery	15	0.32%
Anorectal	11	0.23%
Chinese Medicine Internal Medicine	11	0.23%
Nuclear Medicine	9	0.19%
Infectious Diseases	8	0.17%
Cardiovascular Surgery	7	0.15%
ophthalmology	7	0.15%
Critical Care Medicine	6	0.13%
Neurosurgery	3	0.06%
Physiotherapy Center	2	0.04%
Neonatology	1	0.02%
